# Elimination kinetics of eugenol in grass carp in a simulated transportation setting

**DOI:** 10.1186/s12917-017-1273-3

**Published:** 2017-11-21

**Authors:** Dong-Hao Zhao, Chang-Liang Ke, Qi Liu, Xu-Feng Wang, Qiang Wang, Liu-Dong Li

**Affiliations:** 1 Key Laboratory of Aquatic Product Processing, Ministry of Agriculture, Guangzhou, 510300 People’s Republic of China; 2 Laboratiry of Quality & Safety Risk Assessment for Aquatic Product on Storage and Preservation, Guangzhou, 510300 People’s Republic of China; 3 Fishery Environments and Aquatic Products Quality Inspection & Testing Center of the Ministry of Agriculture, Guangzhou, 510300 People’s Republic of China; 4 South China Sea Fisheries Research Institute, Chinese Academy of Fishery Sciences, Guangzhou, 510300 People’s Republic of China

**Keywords:** *Ctenopharyngodon idella*, Clove oil, Anesthetic, Food safety risk, Drug elimination

## Abstract

**Background:**

Fish are vulnerable to stress from over-crowding during transportation and eugenol is the most common sedative used to minimize fish injury. The ADI value of 2.5 mg/kg is recommended by the Joint FAO/WHO Expert Committee on Food Additives. The aim of this work was to study the elimination kinetics of eugenol following exposure of grass carp to a eugenol bath in a simulated transportation setting.

**Results:**

Grass carp, *Ctenopharyngodon idella* (120 fish) were exposed for 24 h to a 10 mg/L eugenol bath. Sampling was performed during a 96 h period after the 24 h bath. Eight fish were sampled at each time point and muscle, plasma and liver concentrations of the drug were determined by ultra-performance liquid chromatography-tandem mass spectrometry. The concentration-time data of eugenol in each tissue were analyzed using non-compartmental methods. The peak concentrations (C_max_) in plasma, muscle and liver were 7.68, 5.30 and 24.63 mg/kg and the elimination half-lives (t_1/2β_) were 19.79, 10.27 and 55.28 h, respectively. The clearance (CL) values were 0.10, 0.44 and 0.04 L/h/kg and the areas under the concentration-time curve (AUC_0-96h_) were 91.54, 22.44, and 214.12 mg·h/L in plasma, muscle and liver, respectively. After a eugenol exposure bath, drug concentrations in muscle tissue of grass carp were below 1 mg/kg at 8 h and 0.1 mg/kg at 24 h.

**Conclusions:**

The drug concentrations in muscle tissue at 8 h were lower than the recommended ADI value.

## Background

**Fig. 1:**
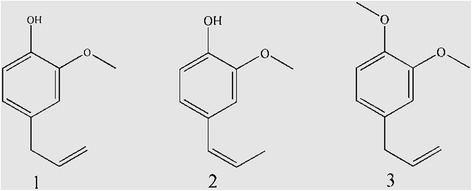
Chemical structures of (1) eugenol (2) isoeugenol and (3) methyleugenol

Eugenol (2-Methoxy-4-(2-propen-1-yl) phenol) and its derivatives isoeugenol and methyleugenol (Fig. 1) are volatile oils extracted from flower buds, leaves and stems of clove, nutmeg, cinnamon, basil and myrcia [1, 2]. The yellowish or colorless liquid has an intense aromatic odor of cloves, is approved as a food additive, and is a Generally Recognized as Safe (GRAS) compound in the United States and European Union [3, 4]. In addition, eugenol has been widely used in dentistry due to its anesthetic and disinfection functions that are active against the bacteria causing gingivitis and dental plaque [5]. Eugenol is also used as a fish sedative because of its fast, stable anesthetic effect and shorter recovery time for freshwater and saltwater fish, although it is not universally approved for this purpose [6].

Fish are vulnerable to stress from over-crowding during long distance transportation and anesthetics can diminish mortality and decrease metabolism [7]. Eugenol is an inexpensive and plentiful anesthetic for fish and is often used to sedate or anesthetize fish in China. However, the use of eugenol and isoeugenol as an anaesthetic for edible fish is controversial.

Australia allows the use of isoeugenol but not eugenol as a fish anaesthetic and the maximum residue limit (MRL) is 100 mg/kg in aquatic products [8]. The relatively new anesthetic Aqui-S that contains approximately 50% isoeugenol is also approved in EU and Norway.

In New Zealand, the MRL of eugenol was 0.1 mg/kg in 2005 but this limit was revoked in 2013 [9, 10]. Eugenol is approved for aquatic medicine as an anaesthetic with an MRL of 0.05 mg/kg in Japan [11]. The acceptable daily intake (ADI) of eugenol at 2.5 mg/kg was recommended by the Joint FAO/WHO Expert Committee on Food Additives (JECFA) [12]. Although the United States FDA lists eugenol as a GRAS additive, they do not consider eugenol to be safe as a fish anaesthetic [13]. The Canadian Council on Animal Care approved eugenol for fish euthanasia but not as an anaesthetic [14].

There are numerous studies focusing on the anesthetic action of eugenol in zebrafish, spotted maigre, *Cynoglossus semilaevis*, *Salmo salar* (Atlantic salmon), rainbow trout, common carp, channel catfish and striped bass [7, 15]. The objective of the current work was to establish the elimination kinetics of eugenol following an exposure bath in grass carp. We wanted to assess the residue risk by simulating eugenol anesthesia during the transportation of live fish.

## Methods

### Animals and reagents

One hundred and twenty grass carp (*Ctenopharyngodon idella*, 1.5 ± 0.1 kg) were purchased from Huanqiu Aquatic Products Market (Foshan, China) and were used in the elimination kinetics experiment. The fish were transported to the lab in a specialized truck and housed in a fiber reinforced plastic tank. Prior to drug exposure, the grass carp were acclimatized and fasted for one week with a continuous flow of aerated tap water. The water temperature was sustained at 25 ± 2 °C and the pH, temperature, ammonia and dissolved oxygen levels were monitored every day during the experimental period.

Eugenol (99.5%) was purchased from Xiashan Aquatic Products Market in Zhanjiang, China. Deuterated eugenol (97.2%) was obtained from Accustandard (USA). The oily liquid was dissolved in aqueous alcohol (analytical grade, 1:10 *v*/v) for use as a tank additive.

### Anesthetic effect of eugenol on grass carp at different concentrations

To acquire a satisfactory anesthetic or sedative effect to study elimination kinetics, we determined the anesthetic activity of eugenol on grass carp at different concentrations. Another thirty grass carp were divided equally into six groups. The fish were medicated with concentrations of eugenol at 5, 10, 15, 20, 30 and 40 mg/L for 8 h and physiological characteristics of breathing rate, body balance and response to stimulation were closely observed. Fish survival was also assessed and the minimum eugenol concentration that kept the fish in deep sedation without mortality was used for the remaining experiments.

### Sample collection

The fish were randomly divided into 15 groups and 8 fish were sampled at each time point. After 24 h exposure bath of eugenol, grass carp were transferred to drug-free water with a net, sacrificed with a blow to the head, and blood, liver and muscle samples were collected at 0, 0.25, 0.5, 1, 2, 4, 8, 12, 16, 24, 36, 48, 72 and 96 h. Blank samples without anesthetic from the fifteenth group were simultaneously collected. Blood samples were collected with anticoagulants and centrifuged immediately at 2000 g for 10 min. Plasma was separated and stored at 4 °C. After rinsing with deionized water to eliminate blood, the tissue samples were wiped with disposable tissues and frozen at −20 °C until analysis.

### Sample preparation

An aliquot of 0.5 mL plasma sample was transferred into a capped 1.5 mL centrifuge tube and mixed with 0.5 mL of acetonitrile. A 0.1 mL aliquot of deuterated eugenol (eugenol-d3) internal standard solution (100 ng/mL) was added and the mixture was vortexed for 30 s and then centrifuged at 12,000 g for 10 min. The supernatant was collected for solid-phase extraction (SPE).

Muscle and liver tissues were homogenized and a 2.00 g sample transferred to a 15 mL polypropylene centrifuge tube and was spiked with 10 ng of eugenol-d3 internal standard solution. After 30 min incubation by standing at room temperature, 5 mL n-hexane was added and the tube was vortexed and then shaken for 5 min. The tube was centrifuged at 5000 *g* for 5 min and the supernatant was transferred into a 10 mL glass-tube. The extraction procedure was repeated and the combined supernatants were evaporated to dryness under a gentle nitrogen stream at 40 °C. The residue was dissolved in 3 mL a 60% methanol–water solution.

The dissolved residue was loaded onto an HLB cartridge (60 mg/3 cm^3^, Waters, Milford, MA) that had been activated with 3 mL methanol and 3 mL water successively. The cartridge was washed with 3 mL 60% methanol–water and eluted with 4 mL methanol. The eluate was concentrated to less than 0.5 mL with a nitrogen stream and adjusted to 1 mL with deionized water. The solution was filtered using a 0.22 μm syringe filter for LC-MS/MS determination.

### LC-MS-MS analysis

The concentrations of eugenol in the plasma, muscle and liver samples were determined by LC-MS/MS. The instrument system was a Waters Acquity Series UPLC equipped with an autosampler, a column heater, a binary pump coupled to a Xevo TQ-S triple-quadrupole mass spectrometer with an electrospray ionization source (Waters, Milford, MA). MassLynx software was used for instrument control and data processing. Gradient elution for LC separation was performed at 35 °C using a BEH C_18_ column (50 mm × 2.1 mm i.d., 1.7 μm particle size) by employing a mobile phase consisting of methanol and water with a flow rate of 0.35 mL/min (Table 1). The injection volume was 10 μL.

**Table 1:** Linear gradient elution program

Time (min)	0	0.5	1.5	2.5	3	4
Methanol (%)	25	25	95	95	25	25
Water (%)	75	75	5	5	75	75

The electrospray ionization source was used in negative mode with the following mass operating conditions: capillary voltage, 2 kV, desolvation temperature at 350 °C with a N_2_ gas flow rate of 700 L/h, cone voltage, 20 V, cone gas flow rate 30 L/h. Qualitative and quantitative analysis of eugenol were carried out using the multiple reaction monitoring (MRM) mode. The protonated molecular ion [M-H]^−^ of 163 was selected as parent ion due to its high abundance in full scan mode. Two characteristic fragment ions of 148 and 121 were identified as daughter ions with appropriate collision energy in product ion scan mode. The ion transitions of 163 → 148 and 163 → 121 were employed in the MRM experiment. The distinctive transition of 166 → 148 for eugenol-d3 that specified the internal standard compound was used for quantitation.

### Elimination kinetics analysis

The concentration-time data of eugenol in plasma, muscle and liver were analyzed using a non-compartmental method using WinNonlin, Version 6.3 software (Pharsight, CA, USA). The area under the plasma, muscle or liver concentration-time curves (AUC) and mean residence time (MRT) were evaluated using the trapezoidal rule with extrapolation to infinity. The elimination or terminal half-life (t_1/2β_) was calculated by dividing 0.693 by λ_z_, where 0.693 is the natural logarithm of 2 and λ_z_ was estimated from the slope of the terminal phase [16]. Clearance (CL) was derived from dose/AUC [17] and volume of distribution in the terminal phase (Vz) was calculated by the equation: Vz = Dose/(λ_z_ × AUC) [18].

## Results

### Physiological characteristics of grass carp in the exposure bath

**Fig. 2:**
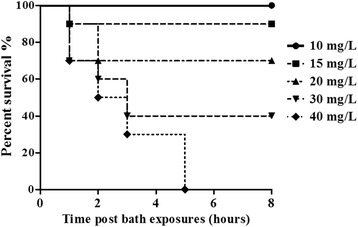
Survival curves for grass carp at various concentrations of eugenol during bath exposure over an 8 h period. Curves were plotted from single experiments using 8 fish at each timepoint

Eugenol induced grass carp anesthesia rapidly with little apparent stress after 10 min exposure to a concentration of 10 mg/L (Table 2). Conversely, mortality was as high as 100% if grass carp were exposed to eugenol levels greater than 40 mg/L (Fig. 2).

**Table 2:** Physiological characteristics of grass carp exposed to eugenol for 10 min at 25 °C

*Eugenol (mg/L)*	*Physiological characteristics*				*Mortality (%)*	*Condition*
	Breathing	Motion	Equilibrium	Response		
*0*	Normal	Normal	Normal	Normal	0	Normal
*5*	Accelerated	Reduced	Normal	Delayed reaction to touch stimuli	0	Sedation
*10*	Reduced	Stop	Normal	No reaction to touch stimuli	0	Deep sedation
*15*	Reduced	Stop	Partial loss of equilibrium	No reaction to touch stimuli	10	Deep sedation
*20*	Reduced	Stop	Total loss of equilibrium	No reaction to touch stimuli	30	Anesthesia
*30*	Reduced	Stop	Total loss of equilibrium	No reaction to touch stimuli	60	Deep anesthesia
*40*	Stop	Stop	Total loss of equilibrium	No reaction to touch stimuli	100	Death

Ten min observation time

We obtained a state of deep sedation when the fish were exposed to a medicated bath containing eugenol at 10 mg/L. The fish could keep their balance with gentle breathing, little activity and no response to touch stimuli. After 8 h exposure bath, they would return to a normal state in several minutes with zero mortality when they were transferred to anesthetic free water. However, five fish died in the elimination kinetics study, and mortality after 24 h was below 5%. Therefore, the ideal anesthetic concentration of 10 mg/L was the optimal dosage and was employed in the following experiments.

### LC-MS-MS analysis

The coefficient of determination (R^2^) for the determination of eugenol levels was above 0.99 in the linear range of 1–500 μg/L. Plasma, muscle and liver samples that were fortified with eugenol at three concentrations of 5, 25, and 100 μg/L (or μg/kg) had recoveries ranging from 89.2% to 96.5%. The limit of quantitation (LOQ) was 0.5 μg/L in plasma and 0.25 μg/kg in muscle or liver sample, respectively.

### Elimination kinetics of eugenol following exposure bath in grass carp

**Fig. 3:**
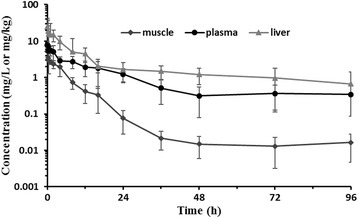
Plasma, muscle and liver concentration-time semilogarithmic profile of eugenol following medicated bath administration for 24 h at a single dose of 10 mg/L (*n* = 8)

No conspicuous paradoxical reactions were observed in grass carp after drug exposure in the bath format. The semi-logarithmic concentration-time plots of plasma, muscle and liver indicated that after a 24 h exposure to 10 mg/L eugenol, the C_max_ were 7.68, 5.30, and 24.63 mg/kg in plasma, muscle and liver, respectively (Fig. 3).

The elimination kinetics were simulated using a non-compartmental model. Compared with plasma and muscle, the drug residue level in liver was noticeably higher. The t_1/2β_ of plasma, muscle and liver were 19.79, 10.27 and 55.28 h, respectively. The CL of plasma (0.10 L/h/kg) and muscle (0.44 L/h/kg) were large compared with the liver (0.04 L/h/kg). The AUC_0-96h_ were 91.54, 22.44, and 214.12 mg·h/L for plasma, muscle and liver, respectively. The AUC_0-96h_ for muscle was only a quarter of that in plasma and one-tenth of that in liver. The mean residence times (MRT) for plasma and liver were 24.25 h and 24.65 h, significantly higher than the 8.50 h for muscle (Table 3).

**Table 3:** Elimination kinetics of eugenol in grass carp following exposure for 24 h at 10 mg/L (*n* = 8)

Parameters	Plasma (mean ± SD)	Muscle (mean ± SD)	Liver (mean ± SD)
C_max_ (mg/L or mg/kg)	7.68 ± 0.31	5.30 ± 0.44	24.63 ± 4.67
AUC_last_ (mg·h/L)	91.54	22.44	214.12
AUC_0-∞_ (mg·h/L)	101.35	22.68	267.29
MRT (h)	24.25	8.50	24.65
CL (L/h/kg)	0.10	0.44	0.04
V_z_ (L/kg)	2.82	6.53	2.98
t_1/2β_ (h)	19.79	10.27	55.28

## Discussion

We determined that the appropriate anesthetic dose of eugenol that produced deep sedation sufficient to transport grass carp was 10 mg/L. However, different fish have a different sensitivity to eugenol. The effective dosages of clove oil for Atlantic salmon, rainbow trout, common carp, channel catfish and striped bass were 10–50 mg/L, 40–120 mg/L, 40–100 mg/L, 100 mg/L and 60 mg/L, respectively [19]. Compared to these fish, grass carp have a high sensitivity to eugenol. The mortality reached 10% if the concentration of eugenol was 15 mg/L (Fig. 2). Therefore, the administered dose of eugenol should be precisely prepared for the exposure bath experiment to avoid producing unsatisfactory anesthetic effects or death.

Drug levels in blood are normally higher than liver or muscle tissues in the absorption and distribution phases. However, in this study we simulated a practical situation in which grass carp were exposed to the drug for an extended period. The drug concentration in the fish body had already reached the steady state by the end of the 24-h exposure. Therefore, the concentration-time data in this experiment actually described the disposition process of metabolism and elimination, even at the first sampling time point. Correspondingly, the concentration of eugenol in liver was higher than blood at 0 h after the 24-h medicated bath. During the 96-h elimination kinetics experimental period, the drug level in liver was always higher than blood, and muscle remained at the lowest drug concentration. These data suggest that the liver is the main organ for eugenol metabolism.

The concentration-time curves of eugenol in plasma, liver and muscle exhibited a monophasic decline, so a non-compartmental model fit the elimination kinetics data well (Fig. 3). Within the first day, levels of eugenol declined quickly in the three sample types. The trend of rapid elimination lasted to the second day for plasma and muscle. However, the elimination rate of eugenol slowed down in each tissue from 48 to 96 h. After drug exposure, eugenol decreased rapidly in liver, from 24.63 ± 4.67 mg/kg to approximately 2 mg/kg at about 12 h, again confirming eugenol is primarily metabolized in the liver.

The C_max_ in muscle tissue was 5.30 ± 0.44 mg/kg, which was one tenth of the C_max_ in rainbow trout after a 100 mg/L exposure for 240 min [20]. The t_1/2β_ of eugenol in muscle tissue was 10.27 h, shorter than in blood and liver. In a previous report, mean concentrations of eugenol decreased from approximately 16 mg/kg to 3 mg/kg within 6 h, and the clearance rate was similar to this work [21]. For liver, a t_1/2β_ of 55.28 h was noticeably different from the methyl eugenol t_1/2β_ of 67.66 min in rats [22]. The species type may have a significant influence on the metabolism and elimination of eugenol. In comparison with mammals, the metabolism velocity of the drug in many fish was slower due to their lower body temperature.

Biotransformation of eugenol leads to the formation of multiple metabolites, such as isoeugenol and methyl eugenol [23]. Their concentrations were determined in another study (Li et al., unpublished data). Methyl eugenol was not detected 30 min after drug exposure. Subsequently, its concentrations in blood, muscle or liver were less than 0.01 mg/kg or mg/L. These residue levels indicated only a small transformation of eugenol methyl eugenol in the grass carp.

On the other hand, the concentrations of isoeugenol were high in grass carp after the medicated bath. However, this metabolite was eliminated rapidly. The residue levels of isoeugenol dropped sharply from the original 5 mg/kg to 0.02 mg/kg in muscle during which time the grass carp recovered from anesthesia (1 h). The compound was not detected at 8 h. On the contrary, the maximum isoeugenol concentrations in blood and liver samples were below 0.5 mg/L or mg/kg, but the residence times was longer. Isoeugenol could not be detected at 72 h in blood and 96 h in liver after recovery from exposure in the eugenol bath. Depletion of isoeugenol from muscle tissue in rainbow trout has been reported but the medicated bath consisted of primarily isoeugenol [24]. Therefore, the residual level of isoeugenol should be also monitored when grass carp are exposed to eugenol.

The JECFA recommended an ADI for eugenol of 2.5 mg/kg. This means that a 70 kg adult ingests less than 175 mg from grass carp if 1 kg is eaten. Fish muscle is the primary edible part of the grass carp and most fish. In the current work, we determined that the concentration of eugenol in muscle at 8 h was below 1 mg/kg. However, due to lack of accurate MRL data, it is not appropriate to declare that aquatic products are safe when fish are anesthetized with eugenol during transportation merely based on this single study. Metabolism and elimination of eugenol in other fish may be significantly different. In addition, we used fasting conditions before eugenol exposure because of the buildup of excrement resulted in turbid water. However, since this might change fat/muscle stores or increase stress to the fish, additional studies are needed to address this issue. The crowding and actual stress of transport were not evaluated, and the safety margin of the drug might be quite different in that setting.

## Conclusions

This work simulated the practical situation of eugenol anesthesia according to the actual usage of this drug during fish transportation procedures. A medicated bath of 10 mg/L eugenol lasting 24 h produced drug concentrations in grass carp muscle that were below 1 mg/kg at 8 h and 0.1 mg/kg at 24 h, it was less than the ADI value recommended by the JECFA.

## Abbreviations

ADI: Acceptable daily intake
AUC: Areas under the concentration-time curve
CL: Clearance
C_max_: Peak concentrations
EFSA: European Food Safety Authority
FEMA: Flavor and Extract Manufacturers Association
GRAS: Generally Recognized as Safe
JECFA: Joint FAO/WHO Expert Committee on Food Additives
MRL: Maximum residue limit
MRT: Mean residence times
t_1/2β_: Elimination half-lives
UPLC-MS/MS: Ultra-performance liquid chromatography-tandem mass spectrometry
Vz: Volume of distribution in the terminal phase
